# Syntax in language and music: what is the right level of comparison?

**DOI:** 10.3389/fpsyg.2015.00942

**Published:** 2015-07-02

**Authors:** Rie Asano, Cedric Boeckx

**Affiliations:** ^1^Department of Systematic Musicology, Institute of Musicology, University of Cologne, Cologne, Germany; ^2^Catalan Institute for Research and Advanced Studies, Barcelona, Spain; ^3^Department of General Linguistics, Universitat de Barcelona, Barcelona, Spain

**Keywords:** comparative cognition, language, music, syntax, action

## Abstract

It is often claimed that music and language share a process of hierarchical structure building, a mental “syntax.” Although several lines of research point to commonalities, and possibly a shared syntactic component, differences between “language syntax” and “music syntax” can also be found at several levels: conveyed meaning, and the atoms of combination, for example. To bring music and language closer to one another, some researchers have suggested a comparison between music and phonology (“phonological syntax”), but here too, one quickly arrives at a situation of intriguing similarities and obvious differences. In this paper, we suggest that a fruitful comparison between the two domains could benefit from taking the grammar of action into account. In particular, we suggest that what is called “syntax” can be investigated in terms of goal of action, action planning, motor control, and sensory-motor integration. At this level of comparison, we suggest that some of the differences between language and music could be explained in terms of different goals reflected in the hierarchical structures of action planning: the hierarchical structures of music arise to achieve goals with a strong relation to the affective-gestural system encoding tension-relaxation patterns as well as socio-intentional system, whereas hierarchical structures in language are embedded in a conceptual system that gives rise to compositional meaning. Similarities between music and language are most clear in the way several hierarchical plans for executing action are processed in time and sequentially integrated to achieve various goals.

## Introduction

Comparative approaches to music and language as cognitive systems have recently gained interest in language and music research, but there seems no general consensus about the fundamental nature of this relationship (e.g., Rebuschat et al., 2012; Arbib, 2013; Honing et al., 2015). The challenge to work out the relationship between music and language from biological perspectives is an ambitious enterprise in cognitive science. It is a tough task because we have to bridge gaps between different research fields, diverse levels of comparison, and distinctive cognitive domains. This issue is the focus of research frameworks investigating biological foundations of language and music called *Biolinguistics* (Boeckx and Grohmann, 2007) and *Biomusicology* (Wallin, 1991; Brown et al., 2000). Though both areas went their separate ways for long time (of course, with some exceptions), recently there is a growing tendency to integrate them to understand the nature of cognitive systems. This is a very fruitful development because both research frameworks seek to answer the same questions: for a given cognitive capacity X: (1) What are the rules and structures involved in X that are necessary to account for what we tacitly know about X?; (2) How does that capacity develop in the individual?; (3) How is X put to use?; (4) How is X implemented in the brain?; How did X evolve? (Boeckx, 2010, p. 187). To give a biologically appropriate explanation to this issue, such proximate research questions (questions about mechanisms and ontogeny) and ultimate research questions (questions about phylogeny and function/survival value) should be investigated (Mayr, 1961; Tinbergen, 1963; Bischof, 2009; Fitch, 2015). Therefore, *Biolinguistics* and *Biomusicology* attempt to integrate theoretical considerations about the capacity for language and music, neuroscientific investigations, and evolutionary considerations. Moreover, biologically appropriate explanations should be obtained by a comparative approach including within-species comparisons such as (developmental) disorders, different cognitive systems (e.g., language, music, and motor cognition) and cultural comparisons, as well as between-species comparisons (e.g., birds, non-human primates, and humans). The research program focusing on this comparative approach is currently called *comparative biomusicology* and *comparative biolinguistics* (Benítez-Burraco and Boeckx, 2014). Finally, within this comparative approach, the often neglected aspect of an ecologically motivated perspective (i.e., social and cultural aspects), should be investigated in light of biology.

In this integrative approach, attention should be paid to the fact that different kinds of *primitives* are explored in linguistics, musicology, neuroscience, and evolutionary research.^1^ This means that a one-to-one transfer of concepts developed for one specific field to the other is problematic. At least, some “linking hypotheses” between different fields and distinctive domains are required. In the current paper, we focus on the most controversial field, namely the inquiry of “syntax” in music and language, and suggest a possible starting point to develop a biologically plausible “mapping hypothesis.” The investigation of syntax is promising from several reasons. First, it is widely accepted that humans are unique in their capacity to combine discrete structural elements in certain (potentially unboundedly) ways to form hierarchically organized sequences. Therefore, revealing the mechanisms of syntax is crucial for understanding the evolution of human cognitive systems. Second, syntax seems to include both domain-specific and domain-general aspects. Investigations of syntax could shed light on the quest for uniqueness of cognitive systems. That is, syntax serves as a window onto the nature of cognitive systems.

**FIGURE 1:**
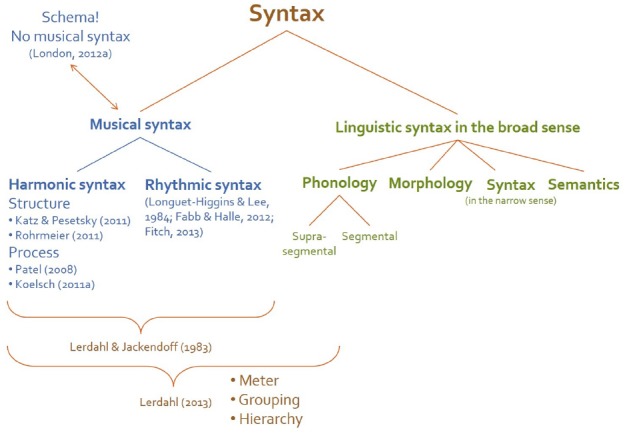
**The broad sense of “syntax” can be investigated at several representational levels (e.g., phonology, morphology, syntax in the narrow sense, semantics, and pragmatics) in language and can be also adapted to music.** In its narrow sense “syntax” is understood as the computational properties at the sentence level, namely the combinatorial principles of words into sentences. Musical syntax includes not only harmonic but also rhythmic aspects (Fitch, 2013). Moreover, it is sometimes claimed that research investigating schema is more promising in examining musical structures instead of syntactic approach because of difficulties to apart structure and meaning in music (London, 2012a).

“Syntax” can be defined as a set of principles governing the hierarchical combination of discrete structural elements into larger units (Bickerton, 2009) and/or into sequences (Patel, 2008).^2^ We take this very general definition as the starting point for the current paper. This broad sense of “syntax” can be investigated at several representational levels (e.g., phonology, morphology, syntax in the narrow sense, semantics, and pragmatics) in language and can be also adapted to music. Musical structure is often considered to be organized hierarchically according to rhythmic and tonal stability. This is why music is investigated in terms of syntax in its broad sense. Tonal encoding of pitch, meter, and grouping are fundamental mechanisms of hierarchical organizations in music.^3^ The broad sense of syntax at several representational levels of music and language are illustrated in Figure 1. In its narrow sense^4^ “syntax” is understood as the computational properties at the sentence level, namely the combinatorial principles of words into sentences. This understanding of syntax is limited to linguistic domain and not fruitful for comparative research. Therefore, we use the term “syntax,” if not marked further, in its broad sense trough out the current paper. The notion of syntax in the broad sense raises two questions which accompany us through the whole article, but cannot be fully answered at the moment: (1) Is syntax best studied apart from semantics? (2) What is the role of syntax in sequence processing (i.e., in performance research)? Our temporary answers are: (1) Syntax and semantics cannot be separated; (2) Syntax accounts for expecting, predicting, and planning future events in a structured way.

Recent comparative approaches investigate syntax of music and language on the several representational levels. In particular, musical syntax is compared to narrow-sense syntax and phonological syntax of language. This one-to-one comparison based on theoretical considerations as well as findings from cognitive neuroscience, however, includes a conundrum: on one hand there are intriguing similarities at both levels of comparison, but there are also important differences. That is, musical syntax does not fit into the ready-made linguistic conception. So, is this distinction of narrow-sense and phonological syntax really useful for investigating syntax of music? Musical syntactic structures are headed hierarchies like linguistic syntax (Jackendoff, 2009), but represent “noncategorical elaborative relations” (Lerdahl, 2013), which means that principles for hierarchical structure building might differ. Moreover, musical syntax cannot be completely separated from structure of musical meaning, namely affect. Given that, as some researchers suggest (Patel, 2008; London, 2012a), syntax of music and language cannot be apart from their meanings which are completely different in each domain, principles governing their structure building cannot be the same. In the first section of the current paper, we provide several reasons why linguistic concepts do not mesh well with findings in music research. The very similarity of music and language lies in the fact that domain-specific hierarchical structures are projected/linearized onto temporal structures (temporal integration).

Thus, the aim of this paper is to find an appropriate level of comparison for the combinatorial properties of music and language, ideally, in a way that is independent of controversies specific to one or the other field. In comparing music and language, specific concepts developed in linguistics are adapted to music, but often prove harmful in the context of comparison. As Dempster (1998, p. 56) already remarked “we should avoid the conspicuously pointless essentialist game of deciding whether music is enough like paradigmatic language to count as a language itself.” The questions are rather “what are the mechanisms of musical syntax?” and “how do they relate to the mechanisms of other cognitive systems?” Patel (2008) also suggested that comparison of music and language should recognize interesting similarities at a more abstract level called “syntactic architecture” of linguistic and musical sequences without trying to adapt specific linguistic concepts to music. Importantly, it should be mentioned that syntax is not a monolithic concept and requires more detailed specification. As Lerdahl (2013, p. 260) suggested, “[c]omparisons of musical and linguistic organization must […] begin at a more fundamental level.” That is, to find the proper level of comparison, “syntax” should be decomposed into basic components, in line with current “divide-and-conquer” approaches in cognitive biology (Poeppel and Embick, 2005; Fitch, 2006, 2010; Boeckx, 2010, 2013; Boeckx and Theofanopoulou, 2014; Boeckx et al., 2014). This is a necessary first step toward future investigations of important questions such as whether syntax is best studied apart from semantics or whether the term “syntax” is appropriate for music research. Such an approach allows us to make the first step toward resolving the conundrum of syntax in music and language.

To find the right level of comparison and adequate granularity of constituents for the analysis, we introduce another level of comparison, namely action. Action-based comparison of music and language is promising mainly from two reasons. First, action is basic component of several cognitive systems including language and music. For example, developmental studies demonstrated the parallel development of word combinations (grammar) and manual object combinations, including tool use (e.g., using a spoon to eat foods) in children (Greenfield, 1991). Action is also involved in speech processing (e.g., articulatory gestures). Moreover, music always involves well-coordinated motor action and is perceived, understood, and interpreted in the way people act upon and interact with music. For example, just hearing music activates several motor areas in the brain (Zatorre et al., 2007; Grahn, 2012). Second, action-based comparison of cognitive systems provides us with an opportunity to consider issue of evolutionary continuity. For example, both humans and apes are species capable of imitating actions of other individuals and using tools, although there are some differences between the imitation and tool capacities in terms of their complexity (Arbib, 2011). Thus, the capacity to process hierarchical structure of music and language might have emerged in course of human evolution on the basis of a more general, evolutionary older function involved in action (Jackendoff, 2009; Boeckx and Fujita, 2014; Fitch and Martins, 2014). That is, investigating the relationship between music, language, and action could provide the first step toward clarifying the nature of syntax in music and language.

In the current paper, based on the ideas introduced by Lashley (1951), we claim that the investigation of temporal integration process and motor program/planning is of particular importance in the inquiry of syntax to resolve the existing conundrum. The conceptual framework we develop in terms of action-related components such as goal of action, action planning, motor control, and sensory-motor integration provides a new possibility for comparative research on music and language from theoretical as well as empirical perspectives. Regarding music and language as parallel to action enables us to explore the syntax of music and language independently of any highly specific linguistic concepts. Language includes two kinds of goals: conceptual goal (organizing thought) and pragmatic goal (communication). While linguistic goals are representational, musical goals are rather experiential and fluid. Music involves affective-gestural and socio-intentional goals. The different properties of goals are suggested to have consequence on the differences between syntax of music and language. Our framework regarding syntax as “a cognitive planning tool” (Baars and Gage, 2010, pp. 390–391) enables us to examine the role of meaning in syntax as well as the account of syntax in music and language processing.^5^ Investigating flexible action planning of music and language in terms of mechanisms, ontogeny, phylogeny, and adaptive significance (Tinbergen, 1963) will reveal the biological foundations of both cognitive systems.

## Conundrums of Syntax

### Two Levels of Comparison: Narrow-Sense Syntax and Phonological Syntax

Ever since *A Generative Theory of Tonal Music* (GTTM) introduced by Lerdahl and Jackendoff (1983) comparisons of hierarchical structures in language and music has looked like a very useful thing to do. This approach, attempting to bridge music theory and psychology, has had a great influence on music research for decades, and we certainly count ourselves among its followers. Though GTTM emphasized the relationship between phonology and music, current comparative research between linguistic and musical structures appear to be more focused on “syntax-syntax” comparison. Thus, some theoretical linguistic approaches have recently suggested that music and language use the same computation for hierarchical structure building (Katz and Pesetsky, 2011; Roberts, 2012). Likewise, Fabb and Halle (2012) claimed that well-formedness of metrical structures in language and music can be determined by the same set of computations, namely grouping and projection, which are regarded as basic syntactic operations of linguistic narrow-sense syntax (Berwick, 2011). The basic idea in this line of work seems to be that the narrow-sense syntactic structures of language is compared with the rhythmic-harmonic structures of music (prolongational structures) which make use of recursively headed hierarchies in which each constituent has a head, and other dependents are modifiers or elaborators of the head (Jackendoff, 2009). Patel (2013) suggested that music lacks to possess “an asymmetry of various types of constructions” (p. 335) such as head-dependent element in language. However, prolongational structure can encode asymmetrical relationship between elements (head-elaboration). Rohrmeier (2011) has gone even further by adapting the hierarchical structure of early generative grammar to Riemann’s functional theory of harmony and regarded tonal functions such as tonic, dominant, and supertonic as parallel to linguistic syntactic categories and called them “functional categories.” At first glance, this narrow-sense syntax comparison of music and language would make sense.

Recently, phonological syntax has been put forward by several authors as a more promising level of comparison between musical and linguistic structures because music and phonology (a domain of speech) make use of combinatorics without meaning (or “syntax without meaning”; Fitch, 2010; Lerdahl, 2013). The differences between the “syntax-syntax” (or syntax in the narrow sense) and phonological syntax are that phonology operates independently of meaning and does not allow self-embedding. “Bare” phonology combines discrete meaningless elements into larger units hierarchically in rule-governed ways (Fitch, 2010). Because combinatorial mechanism in music also includes both properties (i.e., generativity and hierarchy; Patel, 2013), but operates independently of linguistic meaning, the similarity between phonology and music indeed appears to be promising. This proposal is reminiscent of Marler’s (1998; 2000) distinction between phonological syntax/phonocoding and lexical syntax/lexicoding, intended as a fruitful difference for investigating the relationship between animal signaling, language, and music. Marler (2000, p. 36) defined “phonological syntax” as “recombinations of sound components (e.g., phonemes) in different sequences (e.g., words), where the components themselves are not meaningful.” This differs from lexical syntax, defined as “recombinations of component sequences (e.g., words in the lexicon) into different strings (sentences)” (p. 36), which can be regarded as narrow-sense syntax. Learned bird songs, for example, “employ phonocoding to create individual song repertoires numbered in the hundreds” (p. 37). In birdsongs, notes are combined into syllables, “motifs,” and “bouts” (Berwick et al., 2013). This kind of phonocoding can be also found in whale songs—their songs also consists of group/phrases which are recombined into themes (Payne, 2000). That is, phonological syntax/phonocoding is a widespread phenomenon in vocal learning species, and thus provides a promising link to evolutionary research, though there are also some differences between the structure and mechanisms of phonology and song (Yip, 2013). Moreover, the strong relationship between music and speech is emphasized also in developmental research (Trevarthen and Malloch, 2002; Brandt et al., 2012). In considering “syntax” there is tendency to get stuck into the narrow notion of syntax in language, but considering broad notion of syntax could provide deeper insight into similarities and differences of music and language.

### Limitation of Language-Based Explanation for Musical Syntax

The problem of such language-based comparison is, however, that musical syntactic structures, in which several mechanisms such as tonal encoding of pitch, meter, and grouping are interacting, cannot be perfectly aligned with hierarchical structures of language (see Table 1). That is, comparative research on syntax in music and language includes some “gray zones” where similarities and differences exist at the same time. Even though we adapt the broad notion of syntax including phonology, insofar as we adapt specific linguistic concepts to music, the comparison cannot be achieved.

**TABLE 1:** **Overview of similarities and differences of music and language**.

**Levels**	**Features**	**Language**	**Music**
Level of narrow-sense syntax comparison	Syntactic categories	Yes	No
	Propositional meaning	Yes	No
	Lexicon	Yes	No (or very different)
Level of phonological syntax comparison	Pitch Relative pitch	Yes	Yes
	Hierarchy	No	Yes
	Discreteness	No	Yes
	Grouping Hierarchy	Yes	Yes
	Large-scale	No (Yes at the text level)	Yes
	Meter Hierarchy	Yes	Yes
	Isochronicity	No (Yes in poetics)	Yes
	Interaction of pitch, grouping, meter	No	Yes

The similarity between musical and linguistic syntax at the level of narrow-sense syntax comparison boils down to the fact that they can be organized as headed hierarchies (Jackendoff, 2009). Musical headed hierarchy (prolongational structure), in which relative structural importance of the pitch event is determined by rhythmic and harmonic stability, cannot be perfectly aligned with linguistic headed hierarchy based on syntactic categories, propositional meaning, and lexical combinatorial units. In music, first of all, some elements are regarded as *head* and the others as modifiers or elaborations of the *head* based on the relative importance and syntactic categories like (N)oun or V(erb) does not exist (Jackendoff, 2009). Tonal functions (e.g., tonic, dominant, and subdominant) are regarded as “categorical” in some sense (Patel, 2013), but they are “categorical” in the way a certain noun can be interpreted as several syntactic functions (e.g., subject as well as object) depending on its current context and not like the syntactic categories (London, 2012a). Thus, prolongational structures represent “noncategorical elaborative relations” (Lerdahl, 2013). Moreover, there are many musical idioms that do not make use of such a complex pitch structure. Thus, only focusing on tonal functions without considering rhythmic syntax (Fitch, 2013) is not fruitful for the comparison of syntax in music and language.^6^

Second, the relationship between head and elaborations in music define the tension-relaxation pattern of the sequence and thus encodes affect (Lerdahl and Jackendoff, 1983) which is regarded as more directly corresponding with the level of body representation rather than with conceptual structure (Jackendoff, 1987).^7^ Moreover, in considering music as a mode of participatory communication, the difference to meaning in linguistic propositional communication becomes clear: meanings of musical expressions are somehow flexible and not required to be made explicit between performers, listeners, and participants (Cross, 2011). This second aspect is strongly relating to the social function of music, which is not limited to Western music, but widely distributed among different musical cultures (Cross, 2012). Affective and socio-intentional meaning exist also in language (e.g., emotional prosody or pragmatics), but they are just accompanying referential, propositional meaning and thus play a secondary role, while they are primary meanings in music.

Third, music does not possess a rich lexicon comparable to linguistic one defined as a set of linguistic objects stored in long-term memory or a structured list of assemblies of features (e.g., phonological, semantic, and syntactic features; Katz and Pesetsky, 2011). Intuitively, such a rich and stable lexicon cannot exist without a rich conceptual system, which is lacking in music. Even though we may assume the existence of some kind of lexicon in music such as repertoire (Peretz and Coltheart, 2003; Peretz et al., 2009) or a stock of musical formulas stored in long-term memory (Jackendoff and Lerdahl, 2006; Snyder, 2009), this is qualitatively quite different from the lexicon in language.

Similarly, there are many parallels between music and phonology, but differences exist at the same time. Music and speech are learned complex vocalization including pitch, grouping, and metrical structures. In both domains, pitch and temporal structures are rule-based systems. Yet, there are also significant differences. First, “relative pitch processing,” i.e., encoding and recognizing pitch pattern independently of absolute frequency level, is an important shared mechanism of pitch processing in music and speech (Fritz et al., 2013; Patel, 2013).^8^ However, musical pitch processing requires more fine-grained and accurate encoding than pitch processing in speech (Zatorre and Baum, 2012). Moreover, pitches in music are discrete and organized hierarchically (“tonal hierarchy”): every tone is heard in relation to *the most stable tone* called often “tonic” which functions as a cognitive reference point (Koelsch, 2012b; Tillmann, 2012; Patel, 2013). In addition, evidence coming from selective impairment of tonal pitch encoding in (congenital and acquired) amusia (or tone-deafness) supports the uniqueness of pitch processing in music (Peretz, 2006).

Second, on the lower level of perception, the principles of grouping are largely general-purpose gestalt perceptual principles which account for music and speech as well as visual perception in the similar way (Jackendoff and Lerdahl, 2006; Jackendoff, 2009). In music, grouping refers to the hierarchical organization of the musical stream into motives, phrases, and sections (Jackendoff, 2009). Phonological structure also includes a similar kind of process: phonemes are grouped into syllables, which are again grouped into larger phrases. However, beyond this lower level, music makes use of grouping to make sense of larger structures. In a large-scale structure of music, combinatorial primitives are schematic groups rather than pitch events (Lerdahl, 2013). Musical groups at the lower level are combined into larger chunks such as phrases. Contrarily, grouping in phonology is restricted by other linguistic entities like the sentence (Jackendoff, 1987). Intonational phrasing, hierarchical organization of syllables into larger groups, which marks a group boundary, is limited to a sentence and cannot be extended to larger level.

Third, in metrical structures, *beats* are organized hierarchically in metrical grids according to their relative strength. Each note in music and each syllable in speech gets a beat at the lowest level of hierarchy, which is projected onto the higher levels if it is more salient than the others. In this way, the metrical grids of music are formally homologous to those of phonology (Jackendoff and Lerdahl, 2006). However, there are also important differences. In music, “a single note can subtend multiple beats, and a beat can be subdivided by multiple notes” (Jackendoff, 2009, p. 199). This allows for recursive embedding of beats into beats, which is regarded by Longuet-Higgins and Lee (1984) as parallel to phrasal structures of linguistic syntax in its narrow sense. In phonology, on the contrary, one beat is assigned to each syllable and cannot be divided further into smaller units and the complexity of its metrical structure is very limited. Moreover, musical metrical structures are organized isochronously, giving rise to more complex and flexible temporal structures than in speech (Patel, 2008; Jackendoff, 2009).^9^ Empirical evidence for the uniqueness of meter processing in music comes from disorder studies showing that there are not only tone-deaf amusics, but also a beat-deaf case, where the person has difficulties to synchronize to music while his non-musical timing performance is almost intact (Phillips-Silver et al., 2011; Palmer et al., 2014).

Finally, differences become clearer when integrating these subcomponents, namely pitch, grouping, and metrical structures, into more complex hierarchical structures: prolongational structures. Contrary to linguistic prosodic hierarchy, prolongational hierarchy is based strongly on the interaction between pitch and temporal organizations. Moreover, in contrast to phonological rules determining physical changes, musical combinatorial rules have effect on structural aspects (Patel, 2013). As discussed above, several tonal functions can be assigned to the same physical realization (i.e., a tone or chord can be interpreted as tonic as well as dominant depending on its current context). This is the point where the main difference between music and phonology becomes clearer. Prosodic hierarchy also possesses head-like components, namely strong beats, but this relationship doe not encode anything. On the contrary, musical structure encodes tension-relaxation patterning (Lerdahl and Jackendoff, 1983). In this way, musical structure *is* somehow meaningful though this type of musical “meaning” strongly differs from linguistic meaning. This is exactly what makes it impossible for phonological syntax to capture some structural features in music.^10^

In sum, narrow-sense syntax includes many aspects which do not fit to musical syntax, and phonological syntax is not enough for capturing all relevant structural features of music. One question arises: Does the distinction between narrow-sense syntax and phonological syntax make any sense for investigating musical syntax? Sometimes this issue is discussed in relation to the notion of “duality of patterning.” For language, duality of patterning is considered to be a central design feature (Hockett, 1960; Fitch, 2006, 2010; Arbib, 2008): language includes combinatorics of (1) meaningless elements (e.g., phonemes and syllables) into meaningful elements (e.g., morphemes and words) as well as (2) these meaningful elements into larger meaningful units (e.g., phrases and sentences). Because musical structures consist of meaningless elements combined in a meaningful way, the former aspect exists also in music. However, the relation between meaningless elements and “meaning” is less conventionally or arbitrarily determined in music than in language (Bierwisch, 1979). In spite of this difference, it is important to note that both musical and linguistic combinatorics on this level is meaningful.

The difference in the combinatorics on the first level might be no categorical difference between music and language rather a difference of degree, but has an important consequence to the latter aspect of the duality of patterning, namely compositionality. For a structure to be compositional, meaningful units should be primitives of combinatorics. As already discussed above, contrarily to language possessing a rich, stable lexicon in which largely conventionally determined units of “freestanding” meaning, i.e., lexical elements (e.g., words), are stored, it is difficult to find such a unit in music. Moreover, although a stock of musical formulas stored in long-term memory may be thought of as similar to the lexicon in language, they are not the primary primitives of syntactic manipulation (Jackendoff and Lerdahl, 2006). Musical formulas stored in lexicon can be abstract frameworks, patterns, or rules which are modified by composers (and musicians).^11^ For example, an abstract framework such as 12-bar blues can be realized in infinitely variable ways. Therefore, music is claimed not being compositional (Dempster, 1998; Patel, 2013).

Concerning the small-scale level processing, this might be true. However, in a large-scale structure of music, combinatorial primitives are groups (Lerdahl, 2013) and the way in which several groups are relating to each other construct another level of “meaning.” For example, once a theme of the piece is established by grouping mechanism, the thematic relationship can be recognized over the piece, namely in a large scale. That is, the construction of meaningful elements in music takes place in a dynamic way over time and one mechanism that accounts for this aspect is grouping. That is, the evolving representation of meaning, known from the research area of discourse/text comprehension, could be also applied to musical large-scale structure (Seifert et al., 2013). In this way, in a large-scale structure, music is also compositional, but this is quite different from sentence level compositionality of language, rather similar to text level compositionality.

### Temporal Integration—The Very Similarity of Syntax in Music and Language

The hypothesis about shared^12^ neural resources for syntactic integration and different stored representations in music and language was introduced to deal with contradictory evidence from comparative research on music and language: several event-related potential (ERP) and neuroimaging studies tend to provide evidence for commonalities and evidence from cognitive dissociation studies (neuropsychology) tend to support differences and domain-specificity (“shared syntactic integration hypothesis” or SSIRH; Patel, 2003, 2008, 2012, 2013). This lead to the strong emphasis on the shared neural resources for syntax in music and language in the current research. The original idea was based on an ERP study showing that linguistic and musical structural incongruities elicited late positivity, P600 in particular, that were statistically indistinguishable (Patel et al., 1998). The P600 is interpreted as reflecting the integration of representations stored differently in the posterior part of the brain (Patel, 2003). In this experiment, the linguistic incongruities are created on the basis of phrase structure principles and musical incongruities are generated based on principles of harmony and key-relatedness. Patel’s hypothesis is extended as the *Syntactic Equivalence Hypothesis* in Koelsch (2012a) claiming that music-syntactic processing includes hierarchical processing that is shared with other cognitive systems such as language (and action). Especially the early right anterior negativity (ERAN) elicited also by tonal-harmonic violations is regarded as a musical parallel to LAN which indicates morpho-syntactic processing of language (Koelsch, 2011a). In addition, several functional neuroimaging studies using chord sequence paradigms and melodies in which the inferior frontal gyrus (corresponding to BA44) are activated bilaterally with right-hemispheric weighting.

Additional evidence for a close connection between music and language at the level of syntax comes from cognitive disorder studies. Agrammatic aphasics show deficits in harmonic (but not in melodic and rhythmic) syntactic processing (Patel et al., 2008). Children with specific language impairment (SLI) do not show the typical neural indicator for harmonic syntactic processing (Jentschke et al., 2008). Another study with SLI children showed that regular rhythmic prime improves grammaticality judgments (Przybylski et al., 2013). Moreover, one of the abnormalities in the amusic (tone-deaf) brain is the reduced connectivity of right-hemispheric arcuate fasciculus (AF; Loui et al., 2009) connecting the temporal cortex and the right-hemispheric homolog of Broca’s region, which is considered as parallel to the dorsal pathway of speech perception in the left hemisphere, traditionally taken to be the locus of syntactic processing (Friederici, 2012).^13^

Given that the principles of hierarchical structure building in music and language are very different as discussed above, what is actually this shared aspect of syntax in music and language? One possible answer is that shared resources of music and language syntax processing are related to “more general, structural and temporal integration” (Tillmann, 2012, p. 8) which is also involved in linguistic semantic garden-path processing (Perruchet and Poulin-Charronnat, 2013), mathematical processing (Hoch and Tillmann, 2012), cognitive control during stroop task (Slevc et al., 2013), processing of visual narrative structure (Cohn et al., 2014), and action (Sammler et al., 2013). The very similarity of music, language, and those other domains is that they are temporally structured sequences. In processing any sequence, each incoming event needs to be integrated into an evolving representation over time to build structural expectancy. To achieve this, different types of information should be integrated and the relationship between elements should be mapped onto/constructed from linear strings. Therefore, in investigating the problem of syntax in music and language, one important issue that needs consideration is that it mainly deals with the problem of temporal integration, i.e., how stored elements and relations between them are translated into temporal sequences (Lashley, 1951) and how they are constructed from the linear strings. How hierarchical structure being associated with linear string, is currently discussed in terms of multi-step linearization process which is claimed to be carried out within Broca’s area (Boeckx et al., 2014). In processing musical or linguistic sequence, the transition between a multi-dimensional musical or linguistic structure and a one-dimensional linear sequence should take place (Thompson-Schill et al., 2013; Boeckx et al., 2014).

To sum up, the very similarity of syntax in music and language is *the fact that* hierarchical structures bundling different types of information should be mapped onto/constructed from linear strings to make sense of sequences by building structural expectancy by temporal integration. The processes mediating the mapping between hierarchical structures and linear strings might include what is shared in syntax of music and language. To which degree these processes are shared in syntax of music and language is, however, still not clear. Because hierarchical structures of music and language are of different nature as discussed in the section “Limitation of Language-Based Explanation for Musical Syntax,” there might be differences in the process of the temporal integration, as well. Indeed, from empirical side, too, it is claimed that processing higher-order aspects of language and music recruits largely distinct cortical networks, reflecting the different computational building blocks involved in musical and linguistic structure building (Rogalsky et al., 2011). Therefore, to identify the temporal integration as the very similarity of syntax in music and language is a very promising starting point, but this is not enough for a true explanation of their relationship.

## An Alternative: Action—Another Level of Comparison

### Syntax of Action?

To investigate the nature of similarities and differences between syntax of music and language we think that a further domain of comparison might be helpful, namely the comparison between music, language, and action.^14^ There are several reasons why we chose action as the level of comparison. First, action is a hierarchically and temporally structured domain which is the basic component of several cognitive systems including language and music, and is shared to some degree with other species. Thus, it enables us to investigate both proximate questions (mechanisms and development of cognitive systems) and ultimate questions (evolution of cognitive systems). Second, action-based research allows us to explore the relationship between syntax and meaning, especially by considering the role of goals in action planning. Finally, investigating action includes inquiry of mental representations in form of plans as well as the way how they are put into use in company with movement control and sensory-motor systems in a certain context or situation. Therefore, it facilitates the investigation of cognitive systems as situated in a certain environment—this can be examined in terms of flexible planning.

Recently the similarity between action and (narrow-sense) syntax of language receives considerable attention from several fields of cognitive science (Arbib, 2006; Jackendoff, 2009; Koelsch, 2012a; Boeckx and Fujita, 2014; Fitch and Martins, 2014; Pulvermüller, 2014; but Moro, 2014a,b). The strong relationship between these domains are frequently discussed because they share asymmetrical headed hierarchical structures (Jackendoff, 2009) and activate similar brain regions, in particular BA 44 (Fadiga et al., 2009; Fazio et al., 2009; Koelsch, 2011a; Fitch and Martins, 2014; Wakita, 2014). A complex action like making coffee consists of subactions that include more basic subactions (Jackendoff, 2007, 2009). In this hierarchical structure an event made up of a head (the main action), with an optional preparation for the main action, and an optional coda to restore the status quo ante is considered as the basic element (Jackendoff, 2009). Moreover, it is even claimed that single movements might be organized hierarchically (Rosenbaum et al., 2007). Because of these similarities, hierarchical structure of action is sometimes called *action syntax* (Fitch and Martins, 2014; Pulvermüller, 2014) or *action grammar* (Greenfield, 1991; Arbib, 2006; Jackendoff, 2007; Fujita, 2009). That is, there seems to be something shared by syntax of music and language as well as action.^15^ This opens up the possibility of investigating syntax in music and language in terms of action-related components.

The idea to investigate syntax of music and language in terms of action was already introduced by Lashley (1951), who claimed that there should be pre-set or pre-determined units of action (generalized schemas of action^16^) specifying the sequential orders of actions which have no intrinsic order in themselves. For example, concerning speech, individual elements such as words do not determine the temporal series such as sentences. Rather, there should be something else, relatively independent of motor units and thought structure, regulating the order of each sequence. That is, there is something *mediating* motor structure and thought units in temporal sequencing. He identified this as “the problem of serial order” (also “the problem of temporal integration” or “the problem of syntax”), i.e., how stored elements and relations between them are translated into temporal sequences (Lashley, 1951). His main point was that processing of action sequences cannot be explained by a stimulus-response chaining model in which successive responses are triggered by sensory feedback. Apparently, the solution of this problem can be achieved by investigating the nature of generalized schemas or syntax of action. Here, we focus on two relevant conceptions based on Lashley’s idea.

The first one is *motor program*, roughly defined as pre-programming of the movement procedure before actual movement onset (for review, see Summers and Anson, 2009). Lashley’s (1951) notion of the pre-selection of the constituents of action sequences and the pre-determined temporal organization of these constituents was essential for the development of the concept “motor program” (Summers and Anson, 2009). Especially, the notion of “knowing what and how to move before the movement is initiated” is indispensable for successful fast and accurate performance (Summers and Anson, 2009, p. 567). The second concept dealing with the preparing of particular actions is *planning*. This concept is also applied for preparing of behaviors in particular ways in the future (Rosenbaum et al., 2007). While “program” indicates direct involvement of motor activity as its consequence, “planning” is a more abstract concept thought about sometimes also independently of actual execution of movements. In particular (action), planning can be considered as the process of selecting and applying suitable motor programs as well as modifying in a certain situation-specific context.^17^ Both motor program and planning, in spite of this small difference, deal with the existence of pre-determined organization of sequences which can be flexibly modified or adapted according to the current context. This notion of flexible planning system plays a crucial role in our idea of investigating syntax in terms of action-related components.

### Two Central Aspects of Action for Comparative Music-Language Research

#### Hierarchical Plan

One central property of motor program and plan is often claimed to be their hierarchical organization. Especially, the cognitive representational approach to motor control emphasized the notion of hierarchically organized central plans or mental representations in controlling sequences of behavior (Miller et al., 1960; Rosenbaum et al., 2007; Summers and Anson, 2009). As noted above the hierarchical structure is considered as linking action and syntax. The current discussion about the relationship between them, therefore, tend to focus on the notion of the hierarchical plan (Fitch and Martins, 2014). In complex actions, plans consist of subactions which are hierarchically organized in a meaningful way to achieve the goal. Therefore, analogously to language, different goals of action can be understood as different “meanings.”^18^ The way small meaningful units, subactions such as filling the coffee machine with water and coffee as well as turning on the machine, are combined determines the meaning of the complex action, coffee making in this case. Another important aspect in hierarchical organization of action is that goals of actions have influence on the structure of actions. Actions are namely planned in terms of their effects or goals to be achieved (Hommel, 2006). That is, the overall goal of a particular action determines not only constituent elements of the action (subactions), but also the structure of plans. The desired outcome serves as “an invariant representation” or “a reference during planning” (Grafton and Hamilton, 2007, p. 4). This means that differences in goal lead to differences in the way in which actions are planned.

How does hierarchy of action relate to hierarchy of music and language? First, primitives of combinatorics can be compared. Basic actions, movements that achieves some goals, can be regarded as “action words” or “action constructions,” analogs to language words, although this does not mean that action words and language words are completely the same (Arbib, 2006). For example, action words include nothing equivalent to functional words such as “if,” “the,” and “not” (Moro, 2014a). This problem is indeed familiar from the comparison between musical syntax and narrow-sense syntax of language. That is, action and language vary in atomic units of combinatorics as music and language do. Moreover, small meaningful units of action are not “freestanding” in the way language words are. The goals of subactions are determined in terms of the main goal. For instance, putting water into machine without intending to make coffee does not make sense. The word “chair,” on the contrary, possesses own conventionally determined “freestanding” meaning (lexical meaning). The way subactions are meaningful is rather similar to the way musical units get meaningful. Concerning tonal aspect, for example, the tonal center tonic is an anchor for interpreting relationship between pitch events and affect. Of course, there are also conventionalized actions such as gestures, but this is special case in communicative action. However, if we regard lexicon as a stock of formulas stored in long-term memory (as briefly discussed in section “Limitation of Language-Based Explanation for Musical Syntax”), it might be possible to see some similarities between music, action, and language.

#### Sensory-Motor Integration

Another important relationship between music and language (or rather phonology) is the importance of sensory-motor integration (even in the absence of any overt movements). In music and phonology, rule systems are based not only on acoustic features, but also on motor features. For example, the strong coupling of sensory and motor information in speech perception was pointed out by the well-known motor (command) theory of speech perception which claimed that the invariance in phoneme perception arises from articulatory invariance (Liberman et al., 1967), or rather from “the intended phonetic gestures of speaker represented in the brain as invariant motor commands” (Liberman and Mattingly, 1985, p. 2). In music, sensory-motor integration can be best investigated in the domain of rhythm, especially meter, which provides temporal predictability, an intrinsic feature of music driving auditory-motor interactions (Zatorre et al., 2007). This is indicated in the very natural phenomenon that one automatically begins to move or tap the feet along music. Patel and Iversen (2014, p. 1) also emphasize the need of sensory-motor integration for beat perception and propose the “action simulation for auditory prediction” (ASAP) hypothesis: “simulation of periodic movement in motor planning regions provides a neural signal that helps the auditory system predict the timing of upcoming beats.” They also claimed that this sensory-motor integration relies on dorsal auditory pathway connecting auditory and motor regions via the parietal cortex, i.e., connections through superior longitudinal fasciculus (SLF), in particular branch 2 (SLF-II) and temporo-parietal part of SLF (SLF-tp). This is consistent with findings that the functional role of (auditory) dorsal stream is auditory-motor integration, i.e., mapping acoustic speech signals to frontal lobe articulatory networks, involved in speech segment and sequence processing (Hickok and Poeppel, 2007). Notably, this auditory-motor circuit is not limited to speech processing, but is also recruited in perception and reproduction (via humming) of musical sequences including both tonal and rhythmic aspects (Hickok et al., 2003).

The similarity between music and phonology in terms of sensory-motor integration provides further comparative options. BA 44 which is often regarded as a core region of narrow-sense linguistic syntax computing complex hierarchical structure (Friederici, 2012) is also a part of sensory-motor integration circuit for speech processing (Hickok and Saberi, 2012). This is in line with the framework of Brown et al. (2006) developed on the basis of a PET study in which BA 44 is considered as one of sensory-motor centers, respectively, for phonological generativity. Evidences for activity of BA 44 during the sensory-motor integration also comes from several studies on musical rhythm. For example, a PET study found out that BA 44/6 are activated only if the attention of participants was directed to rhythm (Platel et al., 1997). Moreover, BA 44 is also sensitive to violations of rhythmic structure in contrast to isochronic and syncopated rhythm (Herdener et al., 2014).

## Syntax in Terms of Action-Related Components

### A Comparative Framework

**FIGURE 2:**
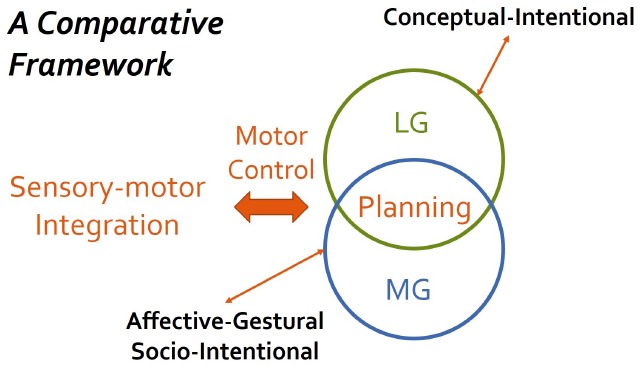
**The syntax of music and language can be investigated within a conceptual framework developed in terms of action-related components such as goal of action, action planning, motor control, and sensory-motor integration.** In this framework, action planning serves as an interface for investigating the relationship between music and language. Moreover, it extends the scope of action-based comparison from functional aspect (what is to be achieved) to more strictly physical aspect (what motions achieve it) by adding further action-related components such as sensory-motor integration and motor control. LG, linguistic goal; MG, musical goal.

Investigating music and language as parallel to action opens the door to resolving the conundrum of syntax. On this level of comparison, the gray zone in narrow-sense comparison can be explained in terms of how hierarchically structured plan is built in order to achieve a certain goal or “meaning” unique to each domain. Hierarchically structured plans of music to achieve musical goals are built in strong relation to affective-gestural system encoding tension-relaxation pattern as well as socio-intentional system, whereas those of language are based on its conceptual structure with rich compositionality and its communicative or pragmatic system.^19^ This could explain why hierarchical structures of music and language differ although they seem similar. Therefore, similarity and difference between syntax in music and language can be investigated by clarifying the way stored representations are integrated and modulated in time within certain contexts by means of building, applying, and adapting hierarchical plans to achieve various domain-specific goals (temporal integration). This will also provide the near insight into the relationship between syntax and semantics. The goal of action serves as the mental reference point for the temporal integration process. In music, mental reference points are mainly set according to tonal hierarchy and the primary beat. Figure 2 shows that action planning serves as an interface for investigating the relationship between music and language. Moreover, it extends the scope of action-based comparison from functional aspect (what is to be achieved) to more strictly physical aspect (what motions achieve it) by adding further action-related components such as sensory-motor integration and motor control. The conceptualization of task goals as well as programming of movements are involved in action research (Jackendoff, 2007; Summers and Anson, 2009).

### Examples from Music Research

#### Brief Remarks on Musical Goals

First of all, because we consider goals as analogous to meaning, a brief discussion about musical meaning is inevitable. While investigating musical meaning, Koelsch (2011b) introduced three kinds of meaning involved in music: extra-musical, intra-musical, and musicogenic meaning. Extra-musical meaning refers to the extra-musical world or concepts and bears some similarity to meaning in language, while intra-musical meaning emerges from “the structural reference of one musical element, or unit, to at least one other musical element, or unit (without reference to the extra-musical world or concepts)” and stands in a strong relation to structural aspects of music (Koelsch, 2011b: 95).^20^ The most important concept is musicogenic meaning, which emerges from the interpretation of effects induced by music. That is, “musicogenic” is used “interpreting “musical meaning” in the sense of significance, the value of music” (Seifert, 2011, p. 122). For example, several forms of affect mentioned above and physical activities such as movement and synchronous movements induced by music are interpreted as having a certain value in affective, esthetic, social, and cultural context. Cross (2011, p. 117) even pointed out that “from an ethnomusicological perspective the dimension of extra-musical meaning scarcely makes sense, as music’s meanings cannot be understood independently of the contexts within which the music occurs.”

The lack of referential, propositional meaning yields the flexibility of musical meaning, i.e., their meanings are not required to be made explicit between performers, listeners, and participants: “Musical meaning is fluid” (Kühl, 2012, p. 123) and can possess the multiplicity of “aboutness” called “floating intentionality” (Cross, 1999, 2010, 2011). This indeterminacy of meaning may arise from three simultaneously existing dimensions of musical meaning (Cross, 2010, 2011): (1) *culturally-enactive* dimension including culturally dependent conceptions of music; (2) *socio-intentional* dimension relating to musical participant’s attitude, communicative or pragmatic stance; (3) *motivational-structural* dimension linking features of the acoustical signal to elicit affect.^21^ Therefore, musical meaning is presentational (Bierwisch, 1979) and experiential (Molnar-Szakacs and Overy, 2006) by being affective instead of being representational and propositional, and facilitates participatory communication rather than propositional communication for exchanging information (Cross, 2011). The emphasis on the social aspect, especially interaction as central to music, beside affective aspects was made by several researchers (e.g., Cross, 2011, 2012; Seifert et al., 2013).

Because of the significant role of affective-gestural as well as socio-intentional meaning, we suggest them as being musical goals. Contrarily to linguistic goals relating largely to conceptual structure, musical goals are relating more to other features of action, namely sensory-motor features. While Mandler (1984) claimed that there is no such structure as a goal in music, Lerdahl and Jackendoff (1983) suggested that musical structure is somehow goal-oriented. For example, in Western tonal music, cadence can be regarded as a kind of structural goal in the dynamics of tension and relaxation. Moreover, concerning social aspect, sharing of temporal experience *as such* can be a goal of music. Below, the first attempt to apply our action-based approach of syntax is briefly introduced with two examples from music research.

#### Affect

As already mentioned above, musical structure encodes affect. Concerning music, “affect” is understood as patterning of tension and relaxation widely existing in human activity and experience (Jackendoff and Lerdahl, 2006). Moreover, it is regarded as an umbrella term of several affective phenomena such as preference, emotion, and mood (Juslin, 2009). Importantly, music possesses two levels of affective structure. On one hand, physical patterns of posture and gesture, often called “musical gestures,” are considered to have direct effect on affective meaning of music (Bierwisch, 1979; Jackendoff and Lerdahl, 2006).^22^ For example, tension-relaxation patterning represented in musical structure is “embodied” in posture and gesture of conductors or musicians, and dance movement. Moreover, increasing tempo may cause tension while decreasing tempo may lead into relaxation. This aspect of affect encoded in musical structure is in such a way directly motivated from musical form.

On the other hand, musical affect also includes partly conventional rules such as tonal grammar or other rules dependent on other idioms or styles. For example, in Western tonal music, tonal hierarchy provides stability conditions serving as a kind of conventional rule determining together with rhythm the structural importance of pitch events, which reflects the tension-relaxation pattern of a musical sequence. Such a joint accent structure is considered to shape structural expectancies of a musical sequence (what will happen when) and would be a basis of affective dynamics (Janata and Grafton, 2003). Such a structural accent can be also created by cadence which can be considered as a goal of tonal motion (Lerdahl and Jackendoff, 1983). This type of musical affect, tension-relaxation pattern reflected in prolongational structure, depends on the listener’s conventionally acquired knowledge of the musical idiom. The units of knowledge which to some degree reflect such structural aspects (e.g., musical phrase structures) are considered as the basis of forming cognitive plans for music performance (Palmer and van de Sande, 1993, 1995).

Both levels of musical affect have very strong relation to movement and bodily representation (Jackendoff, 1987; Molnar-Szakacs and Overy, 2006; Colling and Thompson, 2013). Therefore, Jackendoff (1987) hypothesized that “musical structures are placed most directly in correspondence with the level of body representation rather than with conceptual structure” (p. 239). This is consistent with the idea that “sensory experience of musical pattern is intimately coupled with action” (Janata and Grafton, 2003, p. 682). The former aspect of affect seems to be more directly relating to sensory-motor integration and the latter to action planning. Therefore, the investigation of the dynamic affective processing in music would be a first step toward the explanation of musical syntax—the way how stored representations are integrated and modulated in time within certain contexts by means of building, applying, and adapting hierarchical plans to achieve affective goals.

#### Rhythmic Syntax and Entrainment

Music is a primarily temporal phenomenon and its rhythmic structure is thus an important organizational principle, while linguistic rhythm is more a byproduct of other linguistic phenomena (Patel, 2008).^23^ Nevertheless, rhythmic syntax is often ignored in the current research. Rhythmic syntax accounts for the temporal organization of music, in which discrete structural elements such as beats are hierarchically combined into larger groups according to rules, or grammars, generating well-formed metrical and grouping structures. Though grouping is also one of important mechanisms organizing rhythm, we focus here on meter. As already discussed in the section “Limitation of Language-Based Explanation for Musical Syntax,” musical metrical structures are mainly unique in two ways: (1) hierarchical structure including recursive embedding of beats into beats, which parallels to phrasal structure of language, and (2) isochronicity, permitting more flexible and complex metrical structure than that in speech. The existence of hierarchical structure in meter received recently also support from neuroscientific research (Bouwer et al., 2014). Moreover, it was found that tapping to the main pulse in a polyrhythmic context yielding complex metrical structure creating tension activates BA 47, often considered as one of language area, bilaterally (Vuust et al., 2006).

Rhythmic syntax is appropriate for investigating the relationship between sensory-motor integration, motor control, and action planning from several reasons. First, as already noted, sensory-motor connection is mainly reflected in the domain of meter. Second, there is evidence that the structure of action forms the structure of metrical structure. For example, the way of perceiving pulse in African drumming music is tightly connected to the way to dance (London, 2012b). Third, socio-intentional goal seems to influence metrical structure. In particular, Fitch (2012) claimed that the property of isochronicity emerged because of the need to play together (e.g., in an ensemble). Finally, meter appears to serve as a hierarchical framework for planning and execution of musical events (Mathias et al., 2015).

In particular, these aspects can be investigated in relation to the phenomenon of *entrainment*. In general, “entrainment” refers to “the process by which independent rhythmical systems interact with each other” (Clayton, 2012, p. 49) and can be observed not only in musical context, but also in several biological, physical, and social contexts. In a musical context, the best example is moving to music such as foot-tapping, head nodding, and dancing. In more complex cases, two or more people interact during musical activity such as an ensemble playing and dancing together (Phillips-Silver and Keller, 2012). In such social contexts, actions of individuals (joint actions) are taking place simultaneously. “The experience of music thus involves the perception of purposeful, intentional and organized sequences of motor acts as the cause of temporally synchronous auditory information” (Molnar-Szakacs and Overy, 2006). This is an important difference to linguistic communication in with turn-taking plays an essential role. Musical entrainment requiring auditory-motor integration might reflect musical affective-gestural and socio-intentional goals relating tightly to social aspects to get and belong together and thus partly different than auditory-motor integration in speech (for discussion, see Patel, 2006; Fitch, 2012).

### Implications for Language and Speech Research

In language and speech research, our framework can be applied in a bottom-up fashion, namely in terms of sensory-motor integration and motor control. Phonological rules determine how online movements are produced and controlled. As we saw, musical and phonological rule systems are similar in making use of not only sensory information, but also motor information. The questions how plans relate to motor programs and how planning perspective can be aligned with sensory-motor integration and motor control remain open. One suggestion to relate sensory-motor integration, motor control, and linguistic units was made by Hickok (2012). He introduced a hierarchically organized motor control model (hierarchical state feedback model; HSFC) in which psycholinguistics investigating higher linguistic aspects (speech planning) and motor control approach are integrated. Such an integrative model is promising for the future comparative research on music, language, and action, because differences in music and phonology could also lie in different goals and plans.

## Conclusion

In this paper, we have attempted to find an adequate level of comparison between music and language, to capture the intuition that they share a “syntax.” We saw that many theoretical and experimental investigations tend to focus mainly on hierarchical aspects of musical and linguistic syntax and face the conundrum that similarities and differences exist simultaneously. Musical headed hierarchies based on structural importance regarding rhythmic and harmonic stability cannot be compared in a one-to-one manner with linguistic headed hierarchy based on syntactic categories, propositional meaning, and lexical combinatorial units. The models suggested to resolve this problem in terms of domain-specific representations and shared syntactic integration resources do not make clear how domain-specific representations are activated and integrated by same syntactic resources. Even switching to phonological syntax does not quite solve the conundrum. Mechanisms processing pitch, grouping, and metrical structures seem to be similar in music and speech, but in music pitches are discrete, more fine-grained than those in speech, and hierarchically organized, grouping is less restricted, and metrical structures are isochronous. Moreover, the prolongational structure of music is somehow meaningful in a way that phonological structures are not because it encodes affect. In sum, the very similarity of syntax in music and language is the fact that hierarchical structures bundling different types of information should be mapped onto/constructed from linear strings to make sense of sequences by building structural expectancy by temporal integration. However, this is not enough to explain syntax in music and language.

As a first step toward resolving the conundrum, we introduced another level of comparison, namely action of which the hierarchical organization can be compared to narrow-sense syntax of language, phonological syntax, and musical syntax. We claimed that hierarchical plan as well as sensory-motor integration are of particular importance in the comparative language-music research. The conceptual framework we developed in terms of action-related components such as goal of action, action planning, motor control, and sensory-motor integration provides a new possibility for comparative research on music and language from theoretical as well as empirical perspectives. Regarding music and language as parallel to action enables us to explore syntax of music and language independently of any highly specific linguistic concepts. At this level of comparison, some of the differences between language and music could be explained in terms of different goals reflected in the hierarchical plans: the hierarchical structures of music arise to achieve goals with a strong relation to the affective-gestural system encoding tension-relaxation patterns as well as socio-intentional system, whereas hierarchical structures in language are embedded in a conceptual system that gives rise to compositional meaning. Although we did not discuss the relationship between syntax and semantics in terms of action-oriented perspective explicitly, to us this is a very important research question to be addressed in comparative research on language and music. Especially for musical semantics, an action-oriented approach seems to open up new research perspectives (Seifert et al., 2013). Current research (e.g., Rebuschat et al., 2012; Honing et al., 2015) focuses mainly on comparing syntax of music and language, neglecting the relationship between syntax and semantics. Further theoretical as well as empirical examination is needed in this area. We believe that future comparative research clarifying the role of goals of action, action planning, motor control, and sensory-motor integration in music and language will allow us to gain insight into both the nature of syntax and semantics in music and language as well as the syntax-semantics interface.

### Conflict of Interest Statement

The authors declare that the research was conducted in the absence of any commercial or financial relationships that could be construed as a potential conflict of interest.

## Footnotes

1 Boeckx (2010) discusses this issue more in detail. A comparative approach of language and music in this line of research was introduced by Fritz et al. (2013).

2 “[S]yntax consists of a process for progressively merging words into larger units, upon which are superimposed algorithms that determine the reference of items (in various types of structural configuration) that might otherwise be ambiguous or misleading” Bickerton (2009, p. 11). “[S]yntax in music (just as in language) refers to the principles governing the combination of discrete structural elements into sequences” Patel (2008, p. 241). Though these two definitions are very similar, it is important to pay attention to the difference, namely “larger units” in Bickerton’s definition and “sequences” in Patel’s definition. Whereas “sequence” always includes temporal structure beside abstract and serial structure (Dominey, 2003), “larger units” are not necessarily temporal, i.e., they are atemporal.

3 In Western tonal music pitch is “the most obvious form- and structure-bearing dimension” (Tillmann, 2012). In other musical cultures, however, other aspects such as meter and grouping would play a more important role. For example, in African drum music, meter is the central aspect determining hierarchical structure of music.

4 Some readers familiar with evolutionary research of syntax might think about the distinction between the faculty of language in the narrow-sense (FLN) and in the broad-sense (FLB) introduced by Hauser et al. (2002). Although our distinction here could have something to do with the former distinction, we leave the relationship open in our current paper. For details about this issue, see Hauser et al. (2002), Fitch et al. (2005), Jackendoff and Pinker (2005), Pinker and Jackendoff (2005), and the papers in Larson et al. (2010).

5 Seifert et al. (2013) develop important research ideas for approaching meaning in music, language, and action. Moreover, they pointed out the importance of semiotic perspectives to investigate the relationship between syntax and meaning systems. We do not discuss the relation of these to our current framework explicitly because it is beyond the scope of the current article, but some readers may recognize some parallels between those two approaches.

6 We note here that the importance of rhythmic aspects beside tonal-harmonic aspects was already pointed out in GTTM and subsequent work by Lerdahl (2009, 2013).

7 The existence of the tension and relaxation pattern tends to be regarded as restricted in Western tonal music, but there are good reasons to apply this concept to other musical styles and cultures. For example, in atonal music, in which tonal center is not clear salient conditions based on, e.g., registral prominence, relative loudness, and motivic importance play an important role to build the prolongational structure encoding affect (Lerdahl, 1989). These salient conditions may be adaptable to purely rhythmic music such as African drumming music to construct more complex, sometimes non-isochronous rhythmic hierarchy. Each idiom may possess its own characteristic rules, but the principles of tension and relaxation are considered to be universal (Jackendoff and Lerdahl, 2006).

8 For example, one particular melody, say “happy birth day to you,” in different keys is still recognized as the same melody, intonation contour, and lexical tones can be also identified in different people with different voice frequency. Notably, non-human animals including songbirds have difficulties with this relative pitch processing (Fritz et al., 2013; Patel, 2013). This aspect seems to make human vocalization special.

9 In the linguistic domain, there is also an example where rhythmic aspect becomes an organizational principle, namely poetics (Jackendoff, 1987). In poetic, meter is no more a byproduct of other linguistic phenomena, but comes to possess structural importance on its own. Poetic meter is also isochronously organized.

10 Phonology, especially supra-segmental phonology such as prosody, seems to encode pragmatic meaning. Though there are some approaches investigating the relationship between pragmatics and musical meaning (Chang, 2014), it is still unclear how this comparison can be related to researches on syntax in music and language.

11 Such abstract frameworks are sometimes called schemas. Lerdahl (1991) introduced underlying musical schemas such as phrase and harmonic schemas. For him, such schemas are psychologically accessible and can decompose into a set of features that are instantiated in concrete musical pieces. Further, schemas are suggested to underlie other structural regularities such as meter and standardized musical forms beside tonality and account for musical expectancy building (Snyder, 2009). Note that both Lerdahl (1991) and Snyder (2009) tend to use the term “schema” in terms of cognitive structures which is built through the experience of repeated similar episodes and is involved in expecting types and distributions of events.

12 Some authors (e.g., Brown et al., 2006; Peretz et al., 2015) differentiate sharing, overlap, and parallel conceptually. Although this differentiation is of particular importance in comparative research of language and music, this issue is not discussed in the current paper.

13 It is worth noting that the left-hemisphere bias in speech/language processing is claimed to have a particular importance (Hagoort and Poeppel, 2013). This makes a strong contrast to music showing the right-hemisphere bias in tonal processing (Zatorre et al., 2007).

14 Importantly, action differs from mere movement in including some sort of goal or intention, i.e., dangling arms without having a certain goal or intention is not an action although it is a movement. In this sense, actions (and subactions) include more than just movements that are, however, necessary building blocks of actions.

15 However, it is also claimed that the evidence for Broca’s area as a shared neuronal substrate for human gesture and language is very weak (Barrett et al., 2005). This raises the similar issue discussed above concerning syntax of music and language (Rogalsky et al., 2011). To note that in our framework introduced below we do not claim that syntax of music, language, and action is identical. Rather, we attempt to investigate syntax as such including similarities and differences at the same time. Therefore, several evidences for differences would enrich our framework. For example, Jackendoff (2007) pointed out that action structures are encoded in a different format (e.g., special structure) than that in language.

16 Lashley’s (1951) notion of “schema” is more relating to the notion of motor schema, and thus differs from that of Lerdahl (1991) and Snyder (2009). To note that perceptual and motor schemas can be combined into schema assemblages called coordinated control program (Arbib, 2003).

17 This characterization was developed in the discussion with one of the reviewers and by means of the characterization of “plan” in Jackendoff (2007).

18 A similar idea was already introduced by Koelsch (2012a) and Fitch and Martins (2014).

19 The detailed discussion about the goals in language is beyond the scope of our current article. Here, we would like to briefly note reasons why we chose conceptual and pragmatic goals. Concerning the former, Jackendoff (2007) pointed out that conceptual structure can encode aspects of action. Regarding the latter, Arbib (2006) argued for the importance of communicative aspects for the organization of language. How they are relating to syntax of language is an important issue for the future investigations.

20 The extra-musical meaning might have something shared with propositional meaning of language referring to states of world. However, this plays a secondary role in musical meaning.

21 These three kinds of meaning also exists in language, but they play a more peripheral role in language than in music because of the existence of referential and propositional meaning.

22 For more detailed discussions about “musical gesture,” see Godøy and Leman (2010) as well as Gritten and King (2012).

23 In the linguistic domain, there is also an example where rhythmic aspect becomes an organizational principle, namely poetics (Jackendoff, 1987). In poetic, meter is no more a byproduct of other linguistic phenomena, but comes to possess structural importance on its own.
